# Emergence of NDM-5 Producing Carbapenem-Resistant *Klebsiella aerogenes* in a Pediatric Hospital in Shanghai, China

**DOI:** 10.3389/fpubh.2021.621527

**Published:** 2021-02-25

**Authors:** Fen Pan, Qi Xu, Hong Zhang

**Affiliations:** Department of Clinical Laboratory, Shanghai Children's Hospital, Shanghai Jiaotong University, Shanghai, China

**Keywords:** carbapenem-resistant *Klebsiella aerogenes*, NDM-5, ST4, plasmid, IncX3

## Abstract

**Background:** Carbapenem-resistant *Klebsiella aerogenes* (CRKA) has posed a serious threat for clinical anti-infective therapy. However, the molecular characteristics of CRKA in Shanghai are rarely reported.

**Objective:** This study aimed to investigate the resistance profiles, dissemination mechanism, and molecular characteristics of CRKA strains isolated from children in a pediatric hospital, Shanghai.

**Method:** Fifty CRKA isolates were collected in 2019. Antimicrobial susceptibility of the strains was determined by broth microdilution method. The β-lactamases and outer membrane porin genes were characterized by polymerase chain reaction (PCR). Conjugation experiments were performed to determine the transferability of the plasmids. The plasmids were typed based on their incompatibility group using the PCR-based replicon typing method. Multilocus sequence typing (MLST) and enterobacterial repetitive intergenic consensus-PCR (ERIC-PCR) were performed for the genetic relationship.

**Results:** All CRKA strains showed high level of resistance to cephalosporins and carbapenems, but still susceptible to aminoglycosides, colistin, and tigecycline. Forty five of fifty isolates carried *bla*_NDM−5_ genes (45/50, 90%), alongside with other β-Lactamase genes including *bla*_CTX−M−1_, *bla*_TEM−1_, and *bla*_SHV−11_ being detected. Loss of *ompK35* and *ompK36* genes were observed in 14% (7/50) and 28% (14/50), respectively, with 5 isolates lacking both *ompK35* and *ompK36*. MLST analysis demonstrated that the majority of isolates belonged to ST4 (47/50, 94%) and ERIC-PCR fingerprinting was performed to identify NDM-5-producing isolates with approximately or more than 80% similarity levels. Plasmids carrying *bla*_NDM−5_ were successfully transferred to the *E. coli* recipient and plasmid typing showed that IncX3 were the prevalent among CRKA isolates.

**Conclusions:** Our finding revealed the emergence of NDM-5 producing CRKA belonging to ST4 among children in Shanghai. Further attention should be paid to control the horizontal spread of the Class B carbapenemases like NDM in children.

## Introduction

*Klebsiella aerogenes*, formerly described as *Enterobacter aerogenes*, is an opportunistic pathogen associated with a variety of nosocomial infections including pneumonia, sepsis, and urinary tract infection (1, 2). Emergence of carbapenem-resistant Enterobacterales (CRE) has posed a serious threat for clinical anti-infective therapy and the main resistant mechanism of CRE is production of carbapenemases, although other mechanisms have been proposed, including overproduction of β-lactamases, efflux pumps, porin deficiency, and changes in penicillin-binding proteins. Carbapenemases including *Klebsiella pneumoniae* carbapenemase (KPC), New Delhi metallo- β-lactamase (NDM), and Imipenemase (IMP) have been identified in *K. aerogenes* (3–5).

NDM carbapenemase, as a newly emerging β-lactamase, can efficiently hydrolyse a broad range of β-lactams except aztreonam and has become the most clinically significant due to its increased resistant phenotype and rapid global dissemination. NDM carbapenemases are divided into NDM-1 to NDM-25 variants. To the best of our knowledge, NDM carbapenemases can easily spread among different species of Enterobacterales, with NDM-1 and NDM-5 type being commonly detected. NDM-5 carbapenemase was mainly detected in *E. coli* and rarely in Enterobacterales. However, the prevalence, dissemination mechanism, and clinical characteristics of carbapenem-resistant *K. aerogenes* (CRKA) in Shanghai are still unavailable. In this study, we will focus on CRKA isolated from children in Shanghai, to investigate the resistance profiles and molecular epidemiological characteristics of these strains.

## Materials and Methods

### Study Design and Bacterial Strains

This study was retrospectively performed in Shanghai Children's Hospital, which is one of largest pediatric hospitals in China. A total of 50 non-duplicative nosocomial CRKA strains were collected in 2019. All the isolates were identified by matrix-assisted laser desorption ionization time of flight (MALDI-TOF) mass spectrometry using MALDI Biotyper (Bruker Daltonik GmbHm, Bremen, Germany). CRKA were defined as strains that were resistant to at least one of the carbapenem antibiotics (ertapenem, meropenem, or imipenem) by Centers for Disease Control and Prevention of USA (https://www.cdc.gov/hai/organisms/cre/technical-info.html#Definition). Furthermore, clinical information was obtained from the medical records of each patient. This information included patient demographics, neonatal birth information, brief hospital course, any antibiotic use history of infants, and treatment outcomes, etc.

### Antimicrobial Susceptibility Testing

Antimicrobial susceptibility of all CRKA strains against amikacin, cefuroxime, ceftriaxone, ceftazidime, cefepime, piperacillin-tazobactam, ertapenem, imipenem, meropenem, levofloxacin, trimethoprim-sulfamethoxazole, aztreonam, colistin, and tigecycline were investigated by broth microdilution method. The breakpoints used for interpretation were recommended by the Clinical and Laboratory Standards Institute (CLSI) 2020. The interpretive criterion for tigecycline was based on the breakpoints of the Food and Drug Administration (FDA). Quality control was managed by using *E. coli* ATCC 25922 and *E. coli* ATCC 35218.

### Detection of Resistance Genes

DNA of all strains was extracted by boiling centrifugation method. Polymerase chain reaction (PCR) was used to detected carbapenemase genes (*bla*_NDM_, *bla*_KPC_, *bla*_IMP_, *bla*_VIM_, and *bla*_OXA−48_), ESBL genes (*bla*_CTX−M_, *bla*_TEM_, *bla*_SHV_), AmpC genes (*bla*_DHA_, *bla*_CMY_, *bla*_MOX_, *bla*_EBC_, and *bla*_AAC_) and porin genes (*ompK35* and *ompK36*) as previously described (6, 7). Amplicons were sequenced and nucleotide sequences were further analyzed and compared to sequences available at the National Center for Biotechnology Information (NCBI) website (https://blast.ncbi.nlm.nih.gov/Blast.cgi).

### Multilocus Sequence Typing

MLST analysis was performed for the genetic relationship as described previously (8). Seven housekeeping genes including *dnaA, fusA, gyrB, leuS, pyrG, rplB*, and *rpoB* were amplified and sequenced for *K. aerogenes*. Alleles and sequence types (STs) were assigned at the PubMLST database (https://pubmlst.org/kaerogenes/). Clonal complexes (CC) of these strains were also analyzed.

### Enterobacterial Repetitive Intergenic Consensus-PCR

Furthermore, the clonal relatedness between NDM-5 producing CRKA isolates were investigated by ERIC-PCR using the primers ERIC-Forward (5′ATGTAAGCTCCTGGGGATTAAC-3′) and ERIC-Reverse (5′AAGTAAGGACTGGGG-TGAGCG-3′) (9). Bio-Red Gel Doc system was used to scan gel image and the fingerprinting profiles analysis of these strains was conducted in the BioNumerics software for the similarity dendrogram.

### Conjugation and Plasmid Replicon Typing

To confirm whether the *bla*_NDM−5_ gene was located on plasmids, conjugation transfer experiments were repeatedly carried out by using mixed broth culture method with sodium azide resistant *E. coli* J53 as the recipient. Potential transconjugants that possessed the *bla*_NDM−5_-bearing plasmid were selected on Mueller–Hinton agar plates containing sodium azide (180 μg/ml) and ertapenem (1 μg/ml). The transconjugants were tested for antimicrobial susceptibilities by broth microdilution for testing the above antibiotics. The colonies grown on the selective medium were identified by detecting *bla*_NDM−5_ gene using PCR. Furthermore, PBRT 2.0 kit-PCR-based replicon typing was used for molecular typing of plasmids of transconjugants (10).

### Statistical Analysis

Antimicrobial resistance data was analyzed by using WHONET 5.6. Other statistical data including quantitative data categorical variables was performed by using SPSS 19.0 software (SPSS Inc., Chicago, IL, USA). A value of *P* ≤ 0.05 was considered statistically significant.

## Results

### Clinical Presentation of All Patients

Among 50 patients, most were located at the neonatal department (48/50, 96%) with only two strains from pediatric intensive care unit (PICU). Most of them are male (78%, 39/50) with 22% being female. Most children were <3-months-old, with only two children being 5–months-old and 7-months-old, respectively. The median length of hospital stay was 50 days (6–121 days) and the patients developed CRKP colonization or infection an average of 14 days (2–58 days) after admission. Among 48 patients in neonatal unit, the median birth weight was 1300 g (770–4100 g) and 33 patients were diagnosed as premature infants.

The most common underlying condition among all patients was pneumonia (68%, 34/50). Ninety-two percent of patients had a history of invasive procedures, including central venous catheter, intubation/mechanical ventilation, lumbar puncture, and surgery. Most patients have received more than two antibiotics and β-lactam/β-lactamase inhibitor combinations (74%; 37/50) and carbapenems (64%; 32/50) were the most frequently used antibiotics. Furthermore, a majority of children were improved after treatment during hospitalization and two children were died.

### Isolates Information and Antimicrobial Susceptibility of CRKA Isolates

Among 50 non-duplicated CRKA isolates, 33 (66%) strains were isolated from sputum, followed by tracheal (10/50, 20%), blood (3/50, 6%), swab (3/50, 6%), and urine (1/50, 2%). As shown in Table 1, 100, 98, and 98% of isolates were resistant to ertapenem, imipenem, and meropenem, respectively. Furthermore, all strains were resistant to cefuroxime, cefotaxime, ceftriaxone, cefepime, and piperacillin-tazobactam, while all strains were susceptible to amikacin, gentamicin, trimethoprim-sulfamethoxazole, and tigecycline. In addition, resistance to levofloxacin was seen in 30% of isolates in this study.

**Table 1:** Antimicrobial susceptibility profiles of carbapenem-resistant *Klebsiella aerogenes* isolates.

**Antibiotics**	**Antimicrobial susceptibility, % (n)**		**MIC(μg/mL)**	
	**R**	**S**	**MIC50**	**MIC90**
Cefuroxime	100 (50)	0 (0)	≥32	≥32
Cefotaxime	100 (50)	0 (0)	≥64	≥64
Ceftriaxone	100 (50)	0 (0)	≥64	≥64
Cefepime	100 (50)	0 (0)	≥32	≥32
Piperacillin-tazobactam	100 (50)	0 (0)	≥128/2	≥128/2
Ertapenem	100 (50)	0 (0)	≥128	≥128
Imipenem	98 (49)	2 (1)	≥128	≥128
Meropenem	98 (49)	2 (1)	≥128	≥128
Amikacin	0 (0)	100 (50)	≤2	≤2
Levofloxacin	30.0 (15)	2.0 (1)	1	1
Trimethoprim-sulfamethoxazole	0 (0)	100 (50)	≤1/19	≤1/19
Colistin	–	0(0)	≤0.5	≤0.5
Tigecycline	0 (0)	100 (50)	≤0.5	1

### Distribution of Resistant Genes

Forty five of fifty isolates carried *bla*_NDM−5_ genes (45/50, 90%), which showed that production of NDM-5 carbapenemase was the main mechanism of *K. aerogenes* resistance to carbapenems. Furthermore, loss of *ompK35* and *ompK36* genes were also observed in 14% (7/50) and 28% (14/50), respectively, with 5 isolates lacking both *ompK35* and *ompK36*. Twelve of 45 NDM-5 producing strains examined were deficient in *ompK35* or/and *ompK36*. Among 5 non-NDM-5-producing isolates, *ompK35* or/and *ompK36* were detected in four of them. Moreover, all isolates were found to carry at least one ESBL and/or AmpC gene. Specially, *bla*_CTX−M−1_ group, *bla*_TEM−1_, *bla*_SHV−11_, and *bla*_EBC_ were identified in 96, 96, 56, and 86%, respectively (Table 2).

**Table 2:** Distribution of resistant genes and molecular characteristics of CRKA isolates (No.).

**Characteristics**	**NDM-5 producing CRKA**	**Non-NDM-5 producing CRKA**
Other β-lactamase genes	*bla*_CTX−M−1_(45), *bla*_TEM−1_(45), *bla*_SHV−11_ (26), *bla*_EBC_ (40)	*bla*_CTX−M−1_(3), *bla*_TEM−1_(3), *bla*_SHV−11_ (2), *bla*_EBC_ (3)
Porin genes	*ompK35*(+)+*ompK36*(+) (33) *ompK35*(+)+*ompK36*(-) (7) *ompK35*(-)+*ompK36*(+) (2) *ompK35*(-)+*ompK36*(-) (3)	*ompK35*(+)+*ompK36*(+) (1) *ompK35*(+)+*ompK36*(-) (2) *ompK35*(-)+*ompK36*(+) (2)
MLST	ST 4(45)	ST4 (2), ST199 (1), New STs (2)
Replicon type	IncX3 (45)	/

### Genetic Relationships

**Figure 1:**
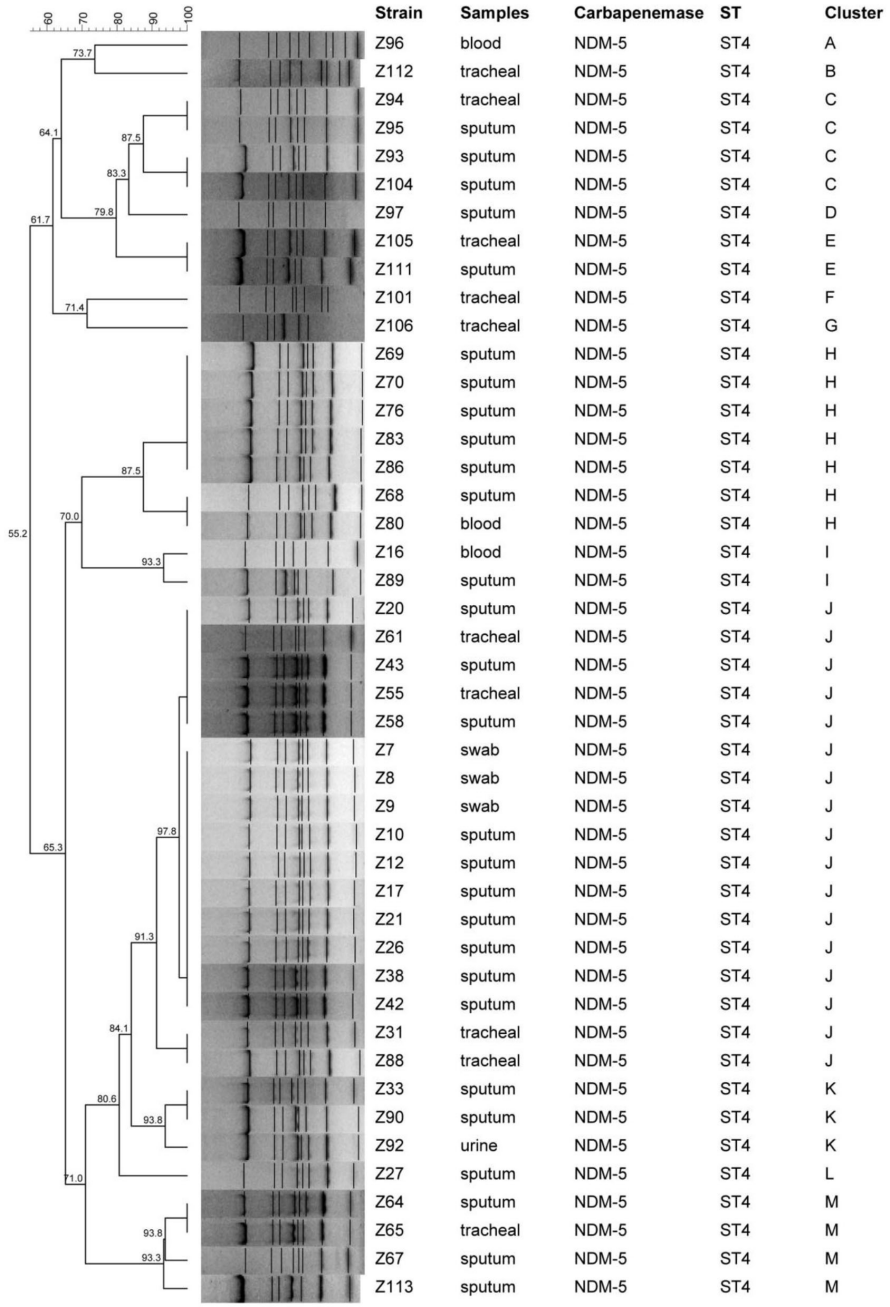
Eric-PCR analysis of carbapenem-resistant *Klebsiella aerogenes* isolates.

MLST analysis demonstrated that these 50 isolates were designed into four different STs and most of isolates (47/50, 94%) belonged to ST4 with allelic profile 3-3-1-4-3-1-2. ST199 and other two new STs were also found in this study. ST4 were identified as CC1. For molecular typing of NDM-5 producing CRKA isolates, a cluster analysis by ERIC-PCR fingerprinting was performed and thirteen distinct ERIC-PCR types labeled as A–M were identified with approximately or more than 80% similarity level, which represented that there existed clonal dissemination among neonatal unit or PICU (Figure 1).

### Conjugation and Plasmid Replicon Typing Analysis

Conjugation experiments revealed that potential transconjugants with plasmids were selected and further identified. The *E. coli* transconjugants exhibited significantly reduced carbapenem susceptibility, and acquired antimicrobial susceptibility pattern similar to those of the donors. In addition, PCR assays demonstrated that all transconjugants harbored the *bla*_NDM−5_ gene and it was successfully transferred from the 45 NDM-5-producing CRKA isolates to *E. coli* J53. According to the PCR-based plasmid replicon typing results, plasmid types of transconjugants carrying *bla*_NDM−5_ belonged to IncX3 (Table 2).

## Discussions

The most common and unequivocal carbapenem resistance mechanism of Enterobacterales involves the production of carbapenemases and this study indicated that NDM-5 was the common carbapenemase in CRKA strains, which was different from other reports conducted in other countries (3, 11). Notably, the detection rate for NDM type carrying Enterobacterales has risen in recent years. NDM-5 type carbapenemase, a variant of NDM-1 with two amino acids substitution (Val88Leu and Met154Leu), was firstly identified in *E. coli* isolate ST648 in United Kingdom from a patient with a previous history of hospitalization in India (12). Then NDM-5 producing strains further spread worldwide, for example, in Europe, United States, Korea, and China, and attracted extensive attention (13–15). Additionally, previous studies showed that NDM-5 carbapenemase has been limited primarily in *E. coli* and *K. pneumoniae*, but rarely in other species of Enterobacterales, although NDM-5 has been found in *Proteus mirabilis* (16), *Morganella morganii* (17)*, Klebsiella quasipneumoniae* (18). Worryingly, NDM-5 producing isolates are also observed in food animals (19) and environment (20) and this phenomenon should be further emphasized.

Despites of the production of carbapenemase, the role of porin deficiency or loss of Omp35 and Omp36 has ever been considered as the main resistant mechanism to carbapenem for *Enterobacter* including *K. aerogenes*, which was previously assigned as a species of *Enterobacter* in 1960. In this study, 16 CRKA strains were detected with loss of *ompK35* and *ompK36* genes and four of 5 non-NDM-5-producing isolates were observed with the loss of *ompK35* or/and *ompK36* genes. However, recently the viewpoint about the role of porin deficiency in CREA was still controversial (21), with the wide spread of carbapenemase among Enterobacterales. Therefore, it is necessary that we should not neglect the importance of porin deficiency, which maybe the main mechanism of non-carbapenemase-carrying CRE, and extensive studies should be further investigated.

The *K. aerogenes* MLST scheme was published in December 2017 (22) and only few studies about molecular characteristics of CRKA all over the world were reported. A previous research presented the detailed description of 91 isolates of *K. aerogenes* with whole-genome sequencing reads available on Genbank and the results showed that the prevalent STs of *K. aerogenes* were ST93 and ST4, belonging to CC3 and CC1, respectively (22). Our MLST analysis suggested that ST4 was the predominant ST type in CRKA strains and there was clonal dissemination among neonates, which was further confirmed by Eric-PCR method. In 2017, we have detected four CRKA isolates from children harboring NDM-5 in our hospital but we did not know the ST of theses isolates (23). Whether the previous isolates are correlated to our study is still unknown and further analysis should be conducted. Therefore, this is the first report of ST4 *bla*_NDM−5_-carrying *K. aerogenes* in children in Shanghai.

To the best of our knowledge, mobile genetic elements such as plasmids are independent of the host genome and responsible for horizontal transfer of resistant genes in Enterobacterales. The *bla*_NDM−5_, encoding NDM cabapenemase, was previously identified in IncX3 and IncFII plasmids (15, 24). Here, *bla*_NDM−5_ gene was demonstrated to be located on IncX3 plasmids and the production of *bla*_NDM−5_-harboring CRKA may be the result of the transmission of IncX3 plasmid from other Enterobacterales, since previous studies revealed that the IncX3 plasmids played a significant role in mediating the widespread of the *bla*_NDM−5_ gene among Enterobacterales, which have been reported in China (23), India (25), and Nigeria (18). IncX3 belonging to IncX-type plasmids always also bear other β-lactamase genes (*bla*_SHV_, *bla*_TEM_, *bla*_ampC_) and other resistance genes (*qnr, sul1*, and *tet*) which contribute to resistance to other antibiotics (17).

In conclusion, this study described the emergence of ST4 NDM-5-producing *K. aerogenes* from children in China and the nosocomial infection of these strain among neonates. The present study also provided the evidence about the clonal dissemination of *bla*_NDM−5_ in *K. aerogenes*. Therefore, it is crucial that urgent and effective measures should be taken to monitor and control the further horizontal spread of the Class B carbapenemases like NDM in children.

## Data Availability Statement

The raw data supporting the conclusions of this article will be made available by the authors, without undue reservation.

## Ethics Statement

The design of the work has been approved by Shanghai Children's Hospital Ethics Committee. This study aimed to obtain the genus and species of bacteria and did not affect the patients. Therefore, our Ethics Committee exempted the patient's informed consent requirements.

## Author Contributions

FP and QX conceived and designed the experiments, performed the experiments, and wrote the paper. FP analyzed the data. HZ contributed reagents, materials, and analysis tools. All authors contributed to the article and approved the submitted version.

## Conflict of Interest

The authors declare that the research was conducted in the absence of any commercial or financial relationships that could be construed as a potential conflict of interest.
